# Characterization of Leader Processing Shows That Partially Processed Mersacidin Is Activated by AprE After Export

**DOI:** 10.3389/fmicb.2021.765659

**Published:** 2021-10-28

**Authors:** Jakob H. Viel, Amanda Y. van Tilburg, Oscar P. Kuipers

**Affiliations:** Department of Molecular Genetics, Groningen Biomolecular Sciences and Biotechnology Institute, University of Groningen, Groningen, Netherlands

**Keywords:** mersacidin, RiPP, lanthipeptide, leader processing, heterologous expression, *E. coli*, subtilisin

## Abstract

The ribosomally synthesized and post-translationally modified peptide mersacidin is a class II lanthipeptide with good activity against Gram-positive bacteria. The intramolecular lanthionine rings, that give mersacidin its stability and antimicrobial activity, are specific structures with potential applications in synthetic biology. To add the mersacidin modification enzymes to the synthetic biology toolbox, a heterologous expression system for mersacidin in *Escherichia coli* has recently been developed. While this system was able to produce fully modified mersacidin precursor peptide that could be activated by *Bacillus amyloliquefaciens* supernatant and showed that mersacidin was activated in an additional proteolytic step after transportation out of the cell, it lacked a mechanism for clean and straightforward leader processing. Here, the protease responsible for activating mersacidin was identified and heterologously produced in *E. coli*, improving the previously reported heterologous expression system. By screening multiple proteases, the stringency of proteolytic activity directly next to a very small lanthionine ring is demonstrated, and the full two-step proteolytic activation of mersacidin was elucidated. Additionally, the effect of partial leader processing on diffusion and antimicrobial activity is assessed, shedding light on the function of two-step leader processing.

## Introduction

**Figure 1:**
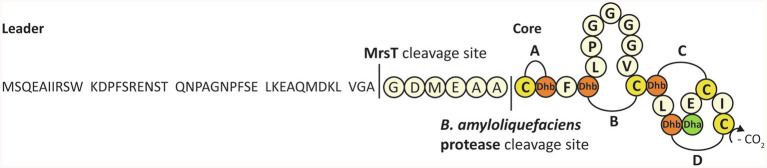
The complete sequence, modification, and processing of mersacidin. The C-terminal cysteine is decarboxylated by MrsD. The four lanthionine rings, A to D, are installed by MrsM. Upon transportation out of the cell, the transporter and leader protease MrsT cleaves a large portion of the mersacidin leader (Viel et al., 2021). The six amino acids of the leader that remain attached to the core peptide keep mersacidin inactive, until they are cleaved off by a *Bacillus amyloliquefaciens* extracellular protease.

The class II lanthipeptide mersacidin is a ribosomally synthesized and post-translationally modified peptide (RiPP; Figure 1; Bierbaum et al., 1995; Arnison et al., 2013). Lanthipeptides contain post-translationally installed intramolecular thioether bridges, which increase resistance to proteolytic degradation and are necessary to give the molecules their rigidity and bioactivity, for example, antimicrobial activity (Repka et al., 2017). The modification enzymes of lanthipeptides and many other RiPPs are guided toward the precursor by a leader peptide sequence (Plat et al., 2013). By combining (parts of) leader sequences of different systems, new molecules can be created that contain modifications from both systems (Burkhart et al., 2017; Wu and van der Donk, 2021). For more in-depth information about the different RiPPs classes and engineering, some excellent reviews are available (Arnison et al., 2013; Montalbán-López et al., 2021).

Like other class II lanthipeptides, mersacidin has intramolecular lanthionine rings that are installed by a LanM (Rahman et al., 2020) enzyme, MrsM (Altena et al., 2000). Additionally, the C-terminal cysteine of mersacidin is decarboxylated by MrsD (Kupke et al., 1994, 1995; Majer et al., 2002). After full modification, the prepeptide is transported out of the cell and its leader peptide is partially cleaved by the bifunctional transporter and protease MrsT (Altena et al., 2000). After mersacidin is activated by an additional processing step in the supernatant, it has antimicrobial activity against Gram-positive strains, including methicillin-resistant *Staphylococcus aureus* (Kruszewska et al., 2004).

Because mersacidin has an unusual and very interesting first ring structure, with potential applications in synthetic biology, a heterologous expression system for the mersacidin biosynthetic genes MrsAMD in *Escherichia coli* has recently been developed (Viel et al., 2021). Using this system, fully modified His6-MrsA (the His-tagged mersacidin precursor peptide) could be obtained. This fully modified precursor peptide could be activated by cleaving the leader peptide using supernatant from the natural producer *Bacillus amyloliquefaciens*. Additionally, it was shown that the bifunctional transporter/leader protease MrsT cleaves the leader only partially, leaving six amino acids of the leader (GDMEAA) attached to the core peptide. GDMEAA-mersacidin is not active until matured by a protease from the supernatant in a second proteolytic step.

Identification of the protease responsible for cleaving the mersacidin leader would greatly improve the previously described expression system in *E. coli*. Cleaving of the mersacidin leader with a single purified protease would allow for a more accurate digestion of any products produced in *E. coli* using the mersacidin biosynthetic genes. Additionally, purification and analysis of digested samples are more straightforward in the absence of compounds from *B. amyloliquefaciens* supernatant. More fundamentally, identification of the mersacidin leader protease is one of the last steps of mersacidin biosynthesis that remains to be described. Finally, because of the unusual ring structure directly downstream of the leader cleavage site, any protease able to cleave at this unusual position might be of interest for synthetic biology and other purposes.

Mersacidin leader processing deviates from that of comparable class II lanthipeptides (Altena et al., 2000; Caetano et al., 2011; Wang et al., 2016). The conserved cleavage site of class II lanthipeptide transporter and leader protease LanT is situated six amino acids before the end of the mersacidin leader (Altena et al., 2000), leading to incompletely processed inactive mersacidin precursor peptide being transported out of the cell (Viel et al., 2021). The function of this incomplete leader processing is not known. It is possible that these amino acids play a role in the maturation or leader processing inside the cell. However, another possibility is that there are advantages of the six amino acids remaining attached to the leader directly after transport, like facilitating transport or increasing diffusion properties. Other possibilities are that mersacidin is only partially processed upon export to protect the producer or that the last six amino acids of the leader play a role in increasing activity as a small peptide after the final processing step.

In this paper, possible proteases involved in mersacidin leader processing are identified by use of readily available *B. subtilis* ATCC 6633 proteases (van Tilburg et al., 2020) and a complementary knockout strain. This approach is feasible due to the relatively high homology between *B. subtilis* and *B. amyloliquefaciens* proteases (Guleria et al., 2016; Contesini et al., 2018). After identification, the candidate mersacidin leader proteases are heterologously produced in *E. coli* in order to improve the heterologous expression system for mersacidin biosynthetic genes in *E. coli*. Finally, several potential functions of the incomplete leader cleavage by MrsT are explored, giving some insight into a potential function of the two-step leader processing.

## Materials and Methods

### Bacterial Strains and Growth Conditions

When grown in liquid medium, all strains used in this study, *B. amyloliquefaciens* BH072*, B. subtilis 168*, *B. subtilis 168* Δ*aprE*, and *B subtilis* PG10, were grown in LB (Formedium) at 37°C at 225 RPM. When pACYC was present, *E. coli* strains were grown with 15μg/ml chloramphenicol, unless stated otherwise. Al transformed strains were grown on LB agar at 37°C. *E. coli* strains containing pACYC were grown with 15μg/ml chloramphenicol. Strains transformed with pDR111 were grown with 100μg/ml ampicillin (*E. coli)* or 50μg/ml spectinomycin (PG10).

### Molecular Cloning

Molecular cloning was performed as previously described (Sambrook and Russel, 2001), adjusted for any conditions specified by reagent manufacturers. The PG10 strain for production of ATCC 6633 AprE-His was available from a previous study (van Tilburg et al., 2020). To construct the PG10 strain for *B. amyloliquefaciens* BH072 AprE-His production, the *aprE* gene was amplified from BH072 with Eco31I restriction-site overhangs and cloned into shuttle vector pDR111 (Supplementary Material S2), amplified by PCR to introduce compatible Eco31I overhangs. The ligated mixture was used to transform CaCl_2_ chemically competent *E. coli* TOP10. For expression in *B. subtilis* PG10, the sequenced plasmid DNA isolated from TOP10 was used to transform *B. subtilis* PG10 (van Tilburg et al., 2020). All cloning for *E. coli* expression purposes was done in *E. coli* TOP10, using the vector pACYC. Using primers to introduce Eco31I restriction-site overhangs, AprE-His from ATCC 6633 was amplified from pDR111-AprE-His, while *B. amyloliquefaciens* AprE was amplified from the BH072 genome. Both fragments were, respectively, cloned into pACYC behind the T7(1) promoter, which was amplified by PCR to introduce compatible Eco31I overhangs, in the case of BH072 AprE introducing a C-terminal His-tag. The mersacidin leader peptide was amplified from pACYC His-MrsA (Viel et al., 2021) and cloned into pACYC behind the T7(1) promoter using a similar method with Eco31I overhangs (Supplementary Material S5). For protein and peptide expression in *E. coli*, sequenced plasmids were used to transform CaCl_2_ chemically competent *E. coli* BL21(DE3).

All oligonucleotides used in this study were obtained from Biolegio (Nijmegen, The Netherlands). PCRs were performed using Phusion (Thermo Scientific) polymerase, which were subsequently cleaned using a “NucleoSpin Gel and PCR Clean-up” kit (Macherey-Nagel). The cleaned PCR products were digested with FastDigest Eco31I (Thermo Scientific), followed by an additional cleaning step. After restriction and cleanup, the respective compatible vector and insert fragments were joined using T4 ligase (Thermo Scientific). This mixture was subsequently used to transform CaCl_2_ chemically competent *E. coli* TOP10. Overnight cultures were made from transformants colonies, after which the Plasmid DNA was isolated using a “NucleoSpin Plasmid EasyPure” kit (Macherey-Nagel) and confirmed by sequencing (Macrogen Europe, Amsterdam, The Netherlands).

### Heterologous Expression

#### *B. subtilis* 168 and *B. amyloliquefaciens* His-AprE Expression in *B. subtilis* PG10

Good PG10 transformants were grown overnight in an Erlenmeyer flask containing 50ml LB. The cultures were diluted to OD_600_=0.075 and grown *ca.* 3h to OD_600_=0.5. Then, expression was induced by the addition of 1mm IPTG. After 4h, the cells were spun down, and the supernatant was harvested.

#### *B. subtilis* 168 and *B. amyloliquefaciens* His-AprE Expression in *E. coli* BL21(DE3)

Per expression, several fresh BL21(DE3) colonies were picked up in LB medium +15μg/ml chloramphenicol and grown overnight. The cultures were diluted 50 times in fresh LB medium containing 10μg/ml chloramphenicol and grown for 2h and 15min. Then, the cultures were cooled down to 16°C in ice water and induced with a final concentration of 1mm IPTG. The induced cultures were grown for 16h at 16°C at 225 RPM, after which the cells were harvested.

#### His-mersacidin Leader Expression

Expression of the His-mersacidin leader was done identical to that of His-AprE up to the point of induction. The expression cultures were kept at 37°C, induced with 1mm IPTG and grown for 4h until the cells were harvested. As a control for the activity test, a similar expression was done using *E. coli* BL21(DE3)+pACYC-*duet* to detect any antimicrobial activity caused by the purification method.

### Peptide Purification

#### His-tag Purification

All His-tagged peptides were purified using 1ml Ni-NTA slurry (Qiagen) in an open column, using manufacturer’s instructions. In case of purification from the cell pellet, the pellet was resuspended in *ca*. 10ml binding buffer (20mm H_2_NaPO_4_ (Merck), 0.5M NaCl (VWR), 20mm Imidazole (Merck), pH 7.4) and sonicated until visible lysed. The sonicated samples were centrifuged for 1h at 10.000 × g, after which the supernatant was loaded onto the column. In case of purification from the expression culture supernatant, supernatant was loaded directly onto the column. After loading, the column was washed with 10 column volumes (CV) binding buffer, followed by an additional wash with five CV wash buffer (20mm H_2_NaPO_4_, 0.5M NaCl, 50mm Imidazole, pH 7.4). Elution from the column was done using 1.8ml 250mm imidazole elution buffer (20mm H_2_NaPO_4_, 0.5M NaCl, 250mm Imidazole, pH 7.4) in case of enzymes and 500mm imidazole elution buffer in case of peptides.

#### C18 Purification

His6-mersacidin leader and the control expression were purified by C18 open column, using 0.25 gram (CV=1ml) of 55–105μm C18 resin (Waters). The 1.8ml His-tag elution samples were acidified using Milli-Q 0.5% trifluoroacetic acid (TFA; Sigma-Aldrich) until a pH of <4 was reached (*ca*. 6ml). The column was wetted using 2 CV acetonitrile (ACN; VWR)+0.1% TFA and then equilibrated using 5 CV Milli-Q+0.1% TFA, after which the acidified samples were loaded. The column was washed with 5 CV 20% ACN+0.1% TFA, after which samples were eluted from the column using 5 CV of 50% ACN+0.1% TFA. The eluted samples were freeze-dried and stored at −20°C until use.

### Antimicrobial Activity Tests

The antimicrobial activity test plates with indicator strain *M. flavus* were prepared as described previously (Viel et al., 2021). All activity tests were performed in petri dishes with a 90mm diameter. In all cases, the positive control consisted of 9μl of 25ng/μl nisin (Sigma) solution. After the samples were spotted, the indicator strain was grown for 24h at 30°C. All activity tests were repeated at least twice.

#### Activation of Mersacidin by ATCC 6633 Proteases

The ATCC 6633 proteases were produced as described previously (van Tilburg et al., 2020). AprE-His was obtained by His-tag purification, Bpr, Epr, and Vpr were used directly from PG10 supernatant, and WprA was used as PG10 cell-lysate. MrsMD modified His6-MrsA was purified by open C18 as described (Viel et al., 2021). Of a 16mg/ml (pre-HPLC) MrsMD modified His6-MrsA solution, 2μl+6.5μl Milli-Q water was digested by 1.5μl of each of the semi-pure proteases, respectively. After 2h of incubation at 37°C, 9μl of each mixture was spotted on an antimicrobial activity plate.

#### Activation of Mersacidin by AprE-His From *B. subtilis* ATCC 6633 and *B. amyloliquefaciens* BH072

For each mixture, 1.5μl of 16mg/ml (pre-HPLC) MrsMD modified His6-MrsA +7.5μl Milli-Q water was digested. For the respective digestions, 1.5μl was added of each of the His-tag purified AprE-His samples. As negative controls 1.5μl of elution buffer (250mm Imidazole) and a His-tag purification of a PG10+empty pDR111 control expression were added.

#### Supernatant Test

All strains were grown for 20h, after which the cells were spun down, and the supernatant was filtered (0.2μm). Of each supernatant, 5μl was added to 2μl 16mg/ml (pre-HPLC) MrsMD modified His6-MrsA or in case of the negative control 2μl Milli-Q water. To each sample, 3μl Milli-Q water was added. All samples were incubated for 2h at 37°C and subsequently spotted.

#### Diffusion Test

Each sample contained 2μl of freeze-dried HPLC purified fully modified His6-MrsA from 1.5 Liter expression culture and dissolved in 200μl Milli-Q water. Then, either 1μl elution buffer (250mm imidazole), 1μl AprE-His (BH072, *E. coli*), or 1μl MrsT150-His (Montalbán-López et al., 2021) was added. The volume of the samples was set to 6μl. All samples were incubated for 3h to assure full digestion of the modified precursor peptides, after which 5μl of each sample was spotted. The digestion efficiency was verified by MALDI-TOF analysis (Supplementary Material S6).

#### Leader Peptide Fragments Test

Each sample contained 1μl fully modified His6-MrsA, 1μl AprE-His (BH072, *E. coli*), and, respectively, 0, 1, 3, or 6μl of freeze-dried His-mersacidin leader or pACYCduet purification control, dissolved in 100μl Milli-Q (Supplementary Material S4). The volume of all samples was set to 10μl with Milli-Q. The samples were spotted after 30min of incubation at 37°C.

### Western Blot

Tricine gels (16%) were prepared as described previously (Schägger, 2006). Two identical gels were run. Of each sample, 10μl His-tag elution +2.5μl 5 × loading buffer (550mm dithiothreitol (Sigma-Aldrich), 250mm Tris-HCl (Boom), 50% glycerol (Boom), 10% sodium dodecyl sulfate (Sigma-Aldrich), 0,5% Coomassie Blue R-250 (Bio-Rad), pH 7.0) was boiled for 5min and the samples were run next to a PageRuler (Thermo Scientific) pre-stained ladder. One of the gels was stained with Brilliant blue stain, and the other gel was used for western blotting (Sambrook and Russel, 2001), using Monoclonal Anti-polyHistidine (H1029, Sigma) as the primary antibody and Rabbit IgG HRP Linked (GENA934, Merck) as the secondary antibody.

### MALDI-TOF Analysis

MALDI-TOF MS was performed as previously described (Zhao et al., 2020). Of digests containing relatively high salt concentrations, that is, digests with His-tagged purified proteases, 0.2μl sample was spotted, after which matrix was added as described.

### MALDI-TOF of Partially and Fully Modified His6-MrsA Digestion With *B. subtilis* ATCC 6633 Proteases

The freeze-dried activatable fraction and non-activatable fraction purified by HPLC, from 1.5 Liter expression volume His-MrsA + MrsM + MrsD (Viel et al., 2021), were dissolved in 500μl Milli-Q water. For each digest, 1μl of respective dissolved peptide or LB medium control and 1μl of the respective protease were added to 8μl Milli-Q water, to a total volume of 10μl. The samples were incubated at 37°C for 1h and analyzed by MALDI-TOF.

### Construction of *B. subtilis* 168 Δ*aprE* Knockout

For the markerless deletion of *aprE* in *B. subtilis* 168, pJOE8999 was used containing the *cas9* gene under control of the *B. subtilis* mannose-inducible promoter (Altenbuchner, 2016). A specific single guide RNA (sgRNA)-encoding sequence to target *aprE* was designed using the CRISPR Guide Design Software of Benchling (*aaagtagctgttatcgacag*) and cloned into pJOE8999 *via* Eco31I digestion. To enable homologous recombination, up- and downstream flanking regions were obtained from the genomic DNA of *B. subtilis* 168 using primer pairs aprE-up-fw + aprE-up-rv (upstream) and aprE-down-fw+aprE-down-rv (downstream). To obtain the final pJOE_ΔaprE vector, flanking regions were digested with SfiI followed by ligation into similarly digested pJOE8999 vector with the sgRNA-encoding sequence. Deletion of *aprE* and loss of pJOE_ΔaprE in *B. subtilis* 168 was achieved as described previously (Altenbuchner, 2016).

## Results

**Figure 2:**
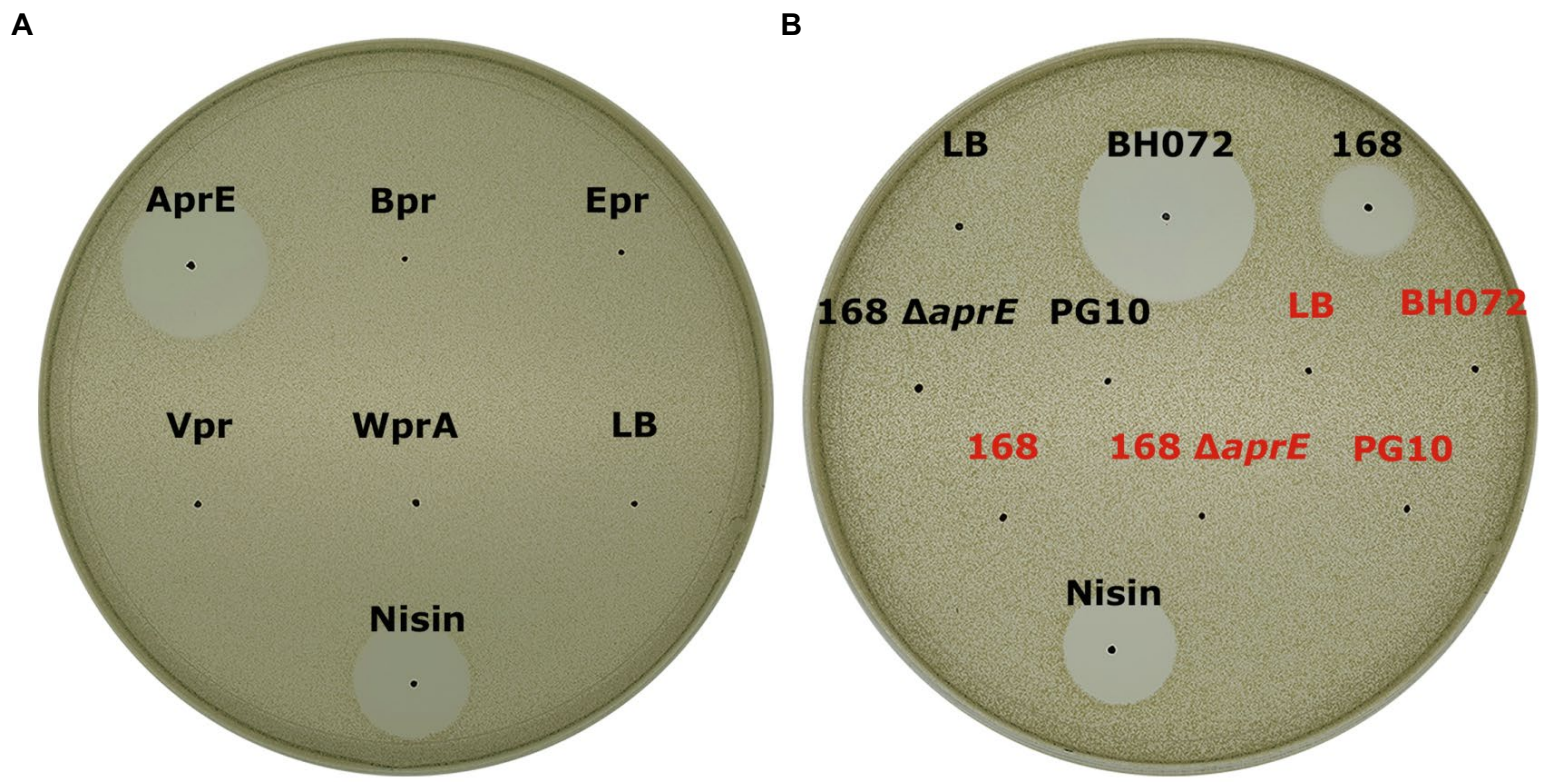
**(A)** Antimicrobial activity test of fully modified His6-MrsA that has been processed by either AprE, Bpr, Epr, Vpr, or WprA against *Micrococcus flavus*. Only AprE-processed fully modified His6-MrsA is activated **(B)** The ability of several overnight culture supernatants to activate mersacidin. The filtered supernatant of *B. amyloliquefaciens* BH072, *B. subtilis 168*, *B. subtilis* 168 Δ *aprE*, and *B. subtilis* PG10 was incubated with either fully modified His6-MrsA (black) or an equal volume of Milli-Q water (red) and spotted against *M. flavus*. The BH072 supernatant had by far the highest efficacy at activating mersacidin, although 168 supernatant is also able to activate mersacidin. Interestingly, the *aprE* knockout strain of *B. subtilis* 168 was no longer able to activate mersacidin, meaning AprE is the only protease produced by *B. subtilis* 168 that can process the mersacidin leader.

To identify the proteases in *B. amyloliquefaciens* BH072 supernatant responsible for the activation of mersacidin, five readily available semi-pure *B. subtilis* ATCC 6633 proteases, that is, AprE, Bpr, Epr, Vpr, and WprA (van Tilburg et al., 2020), were used to cleave (pre-HPLC) MrsMD modified His-6 MrsA *in vitro*. The digested modified His-6 MrsA + protease mixtures were spotted on an antimicrobial activity plate, using *Micrococcus flavus* as the indicator strain. The digested prepeptides were also analyzed by MALDI-TOF. Of the tested proteases, only AprE (subtilisin) was able to activate mersacidin (Figure 2A). Surprisingly, MALDI-TOF analysis of the inactive digests by the other tested proteases also showed products resembling the mass of mersacidin, which is explored and explained later (Supplementary Material S1).

**Figure 3:**
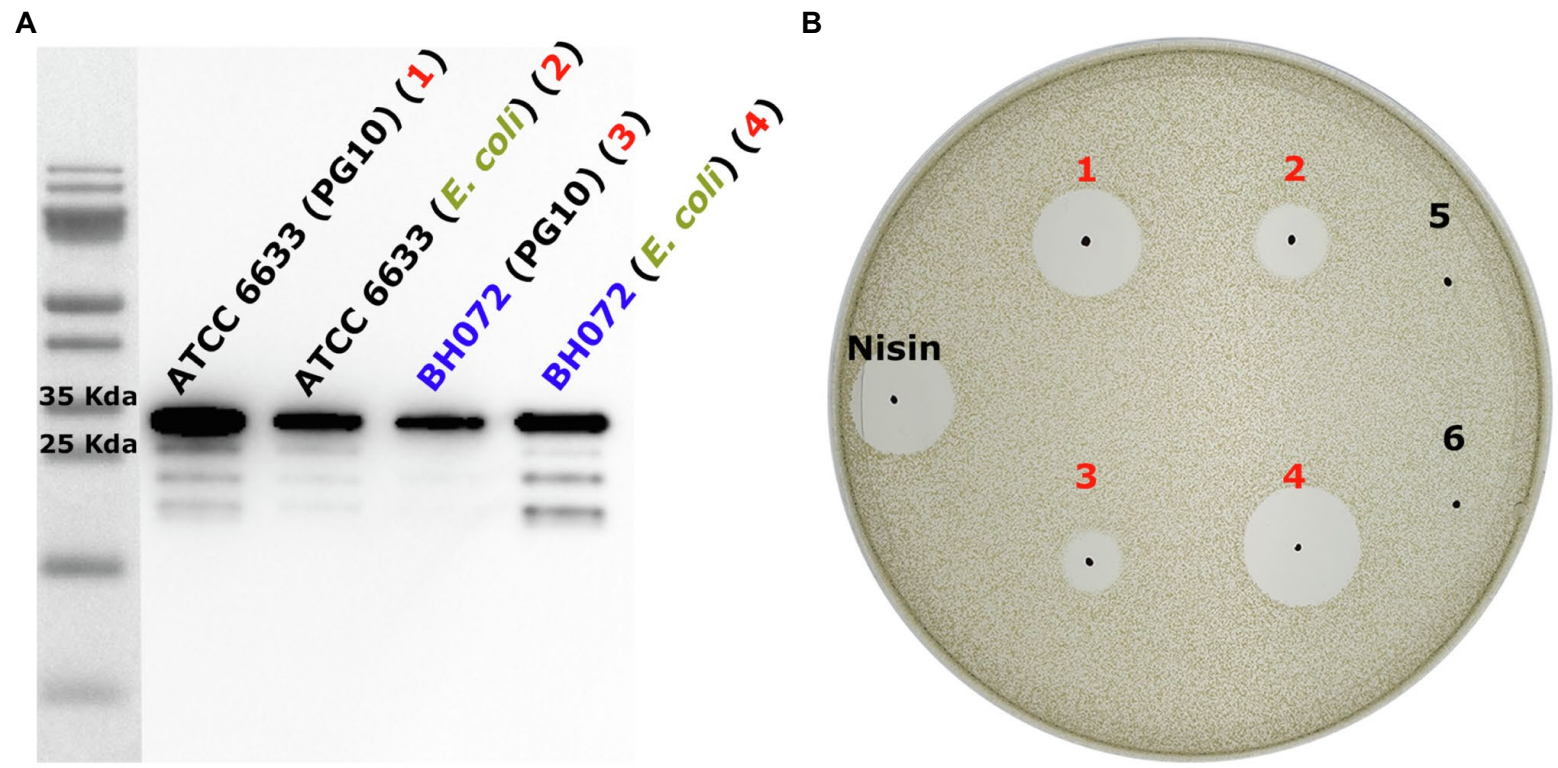
**(A)** Anti-Histag western blot of heterologously expressed AprE-His from ATCC 6633 and BH072 in either *B. subtilis* PG10 or *Escherichia. coli* BL21(DE3). All AprE-His variants are located on the gel at their approximate autoprocessed size, 28655.3Da (ATCC 6633) and 28370.1Da (BH072; Supplementary Material S2). While ATCC 6633 AprE-His is expressed very well in PG10, the expression level of BH072 AprE-His in this strain is much lower. The expression level of both AprE-his variants in *E. coli* BL21(DE3) is equally high and of an average level when compared to the expression levels in PG10 **(B)** The four produced AprE-His samples were used to cleave fully modified His6-MrsA in an antimicrobial activity test against *M. flavus*. Comparing both the PG10 produced ATCC 6633 AprE-His (1) and the BL21(DE3) produced ATCC 6633 AprE-His (2) to BL21(DE3) produced BH072 AprE (4), it is clear that BH072 AprE cleaves the mersacidin leader at a much higher efficiency than ATCC 6633 AprE. It is possible that BH072 AprE-His production in PG10 can be increased by changing the signal peptide sequence to that of ATCC 6633 AprE. Negative control samples 5 and 6 are, respectively, digested with an empty plasmid control expression and His-tag elution buffer.

To verify that AprE from *B. amyloliquefaciens* is indeed the protease responsible for activating mersacidin, the *B. amyloliquefaciens aprE* gene homolog (85% sequence similarity; Supplementary Material S2) was amplified from the BH072 genome and expressed with a C-terminal His-tag in mini*Bacillus* PG10. Simultaneously, the C-terminally His-tagged AprE genes from both ATCC 6633 and BH072 were, respectively, cloned into *E. coli* vector pACYC and produced in *E. coli* BL21(DE3). AprE-His expressed in both PG10 and BL21(DE3) was purified by Ni-NTA affinity chromatography, in PG10 from the supernatant, and in BL21(DE3) from the cell pellet. The AprE homologs from both *Bacillus* strains were able to activate mersacidin, either produced in BL21(DE3) or PG10 (Figure 3). The approximate yields of the AprE variants were quantified by western blot (Supplementary Material S3). BH072 AprE produced in *E. coli* has a higher activity than the PG10 produced ATCC 6633 AprE, despite having a lower yield. This effect could be caused by BH072 AprE cleaving the mersacidin leader more efficiently or by the different expression conditions used for PG10 and BL21(DE3). The ability of BH072 AprE to activate mersacidin confirms that this is a major protease responsible for activating mersacidin in natural conditions.

A *Bacillus subtilis* 168 Δ *aprE* strain was used to confirm that no other proteases from *B. subtilis* can activate mersacidin. MrsMD modified His6-MrsA was digested with the supernatant of multiple *Bacillus* strains, that is, *B*. amyloliquefaciens BH072, *B. subtilis* 168, *B. subtilis* 168 Δ *aprE*, and PG10. While *B. subtilis* 168 supernatant could activate mersacidin, the *aprE* knockout strain supernatant was unable to (Figure 2B). The supernatant of *B. amyloliquefaciens*, however, was able to activate mersacidin at a better rate than that of *B. subtilis* 168. It is possible that AprE from *B. amyloliquefaciens* cleaves the leader more efficiently, more AprE is produced by *B. amyloliquefaciens*, or that other proteases from *B. amyloliquefaciens* can help to activate mersacidin as well.

**Figure 4:**
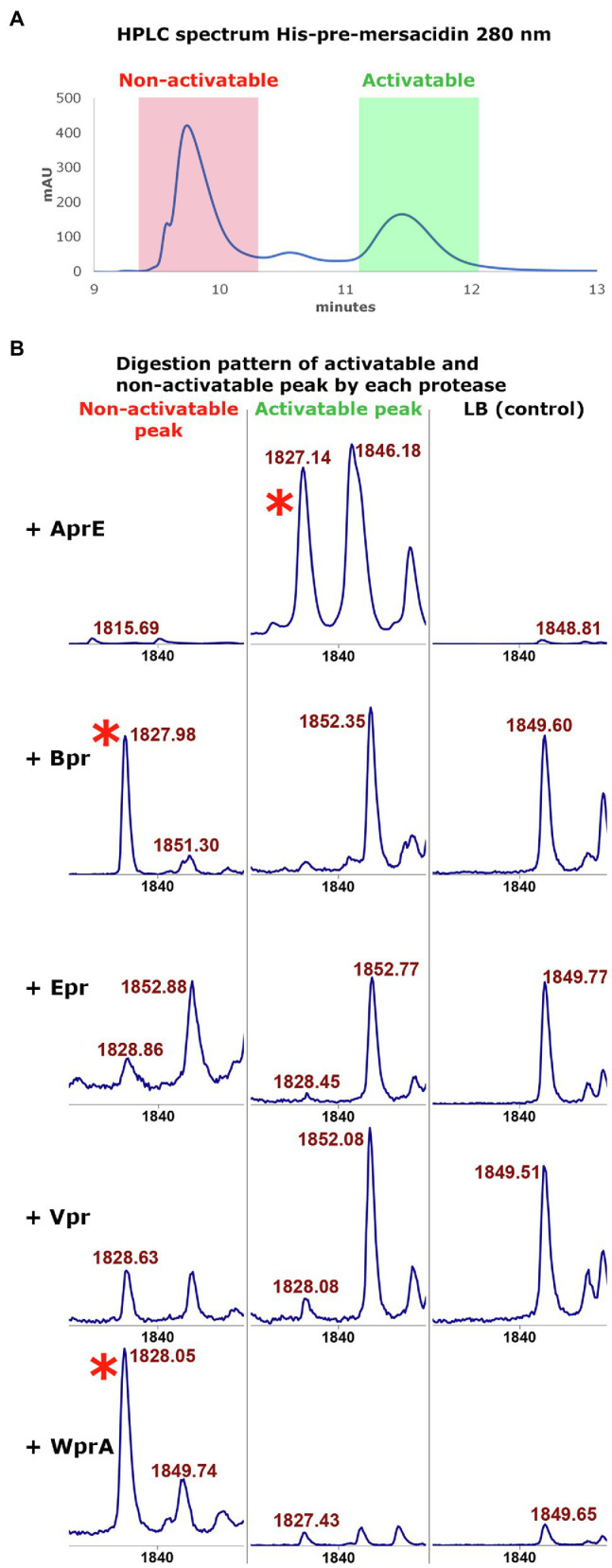
**(A)** The HPLC spectrum of modified His6-MrsA (280nm) shows two distinctive peaks. The peak with the lower retention time (red) contains only partially modified His6-MrsA, which has no antimicrobial activity upon FIGURE 4 |  removal of the leader peptide. The peak with a higher retention time (green) contains fully modified His6 MrsA, which can be activated by removing the leader peptide. Both peaks were collected to use in digestion experiments with the ATCC 6633 proteases. **(B)** The activatable and non-activatable peak were each digested, respectively, with AprE, Bpr, Epr, Vpr, and WprA. The activatable peak digested with AprE has antimicrobial activity and produces a peak with the mass of mersacidin, mersacidin + Na and mersacidin +K (1826Da, 1848Da, and 1865Da (Herzner et al., 2011), red asterisk). However, the non-activatable peak also produces a large peak of 1826Da when digested by Bpr and WprA that lacks antimicrobial activity. Since Bpr and WprA do not produce this peak when digesting the fully modified His6-MrsA, formation of ring A probably inhibits proteolytic activity by these proteases. This suggests that a fraction of the non-activatable peak, while fully dehydrated, lacks the first ring. Absence of ring A then allows the other proteases to cleave the mersacidin leader at a higher efficiency, releasing inactive mersacidin.

A remaining question was why a peak with the mass of mature mersacidin was observed upon digestion with all tested ATCC 6633 proteases, even though only AprE could activate mersacidin in the antimicrobial activity tests. During the purification of fully modified His6-MrsA by HPLC, two peaks were observed. Although these peaks show similar mass distributions by MALDI-TOF analysis (Viel et al., 2021), only one of them contains activatable mersacidin (Figure 4A). Most likely, the peak containing non-activatable mersacidin is fully dehydrated, yet lacks the formation of one or more rings.

To test this hypothesis, the activatable and non-activatable peak were separated by HPLC and subsequently cleaved by each of the semi-pure *B. subtilis* 168 proteases, respectively, (Figure 4B). It was found that Bpr, Epr, Vpr, and WprA do not produce a large peak with the mass of mersacidin after digesting the activatable peak. However, upon digestion of the non-activatable fraction, especially Bpr and WprA produce large peaks with the mass of mersacidin [1826Da (Herzner et al., 2011)]. These results indicate that the first ring is not formed in a part of the product in the non-activatable fraction. The presence of the first ring thus likely prevents proteases, other than AprE, from cleaving the mersacidin leader. Vpr and WprA appear to be able to cleave the activatable peak to some extent, but to not release enough mersacidin for measurable activity. It thus appears that from the tested proteases, AprE is uniquely able to fit the first ring of mersacidin in its active site. Since AprE is also easy to produce in both *Bacillus* and *E. coli*, this find is an interesting and valuable addition to the *E. coli* expression system for mersacidin biosynthetic genes.

**Figure 5:**
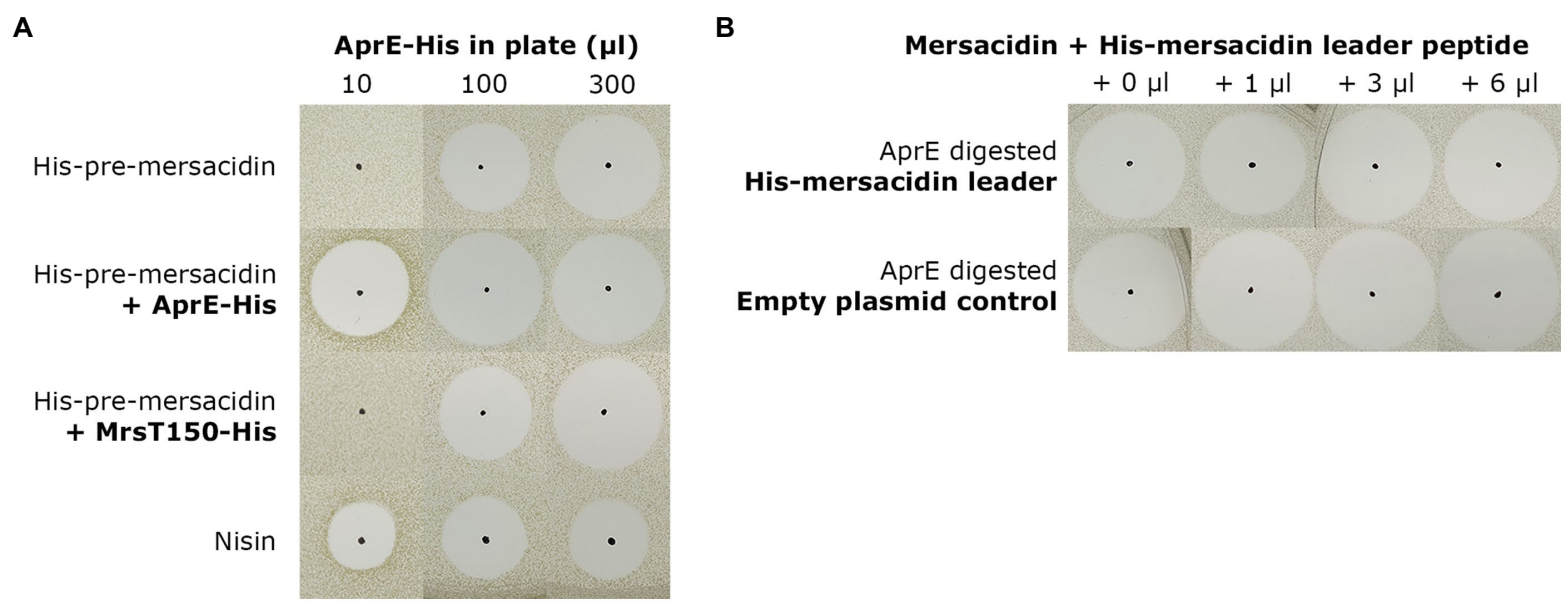
**(A)** To assess the effect of partial leader processing on the diffusion properties of mersacidin, undigested, AprE-His digested, and MrsT150-His digested fully modified His6-MrsA was spotted on bacterial lawns containing AprE-His. The digested samples were spotted on three plates, containing 10μl, 100μl, and 300μl AprE-His (Supplementary Material S3), respectively, and checked by MALDI-TOF analysis (Supplementary Material S6). Undigested and MrsT150-His digested fully modified His6-MrsA clearly have lower activity in the plates with 10μl and 100μl AprE-His, and at best equal activity in the plate containing 300μl AprE-His. The partially processed mersacidin does not show better diffusion properties compared to fully digested mersacidin, only matching fully processed mersacidin when approaching an excess of AprE-His in the plate. **(B)** To test the effects of, fractions of, the mersacidin leader on mersacidin activity, it was heterologously expressed in *E. coli*. And, fully processed mersacidin was purified by C-18 open column after digestion with AprE-His. Then, a constant amount of mersacidin was supplemented with increasing concentrations of AprE-digested His-mersacidin leader. As a control, and empty plasmid expression control was used which was subjected to an identical purification protocol. No effects on antimicrobial activity could be observed in the presence or absence of fractions of the mersacidin leader, suggesting it plays no role after mersacidin is transported out of the cell.

Finally, two tests were performed to explore possible effects of the incomplete leader removal upon its transportation out of the cell by MrsT. The N-terminal 150 residue proteolytic domain of MrsT, responsible for partial leader cleavage upon export, was expressed with a C-terminal His-tag (MrsT150-His) as described previously (Viel et al., 2021). To test the effect of incomplete leader cleavage on the diffusion properties of mersacidin, identical amounts of undigested, AprE-His digested, and MrsT150-His digested fully modified His6-MrsA were spotted on an antimicrobial activity plate containing purified *B. amyloliquefaciens* AprE-His (Figure 5A). It was found that the six amino acids of the leader do not increase diffusion properties, but rather hamper the activation of mersacidin when there is no excess of AprE.

Next, the possibility that the leader, or parts thereof, play a role in enhancing mersacidin activity was explored. A gene encoding just the His-leader peptide sequence of mersacidin was cloned into pACYC and expressed in *E. coli* BL21(DE3; Supplementary Material S4). Then, mature mersacidin was purified from AprE-digested fully modified His6-MrsA by open C-18 column. The purified mature mersacidin was spotted on an antimicrobial activity plate with or without the presence of AprE-digested mersacidin leader peptide (Figure 5B). Results show that the leader, or fragments of the leader, do not enhance antimicrobial activity. The results of the latter two experiments suggest that the last six amino acids of the leader peptide have no significant role after the peptide has been matured and transported out of the cell, although they might facilitate secretion (opposed to secretion with the whole leader attached) and by keeping the peptide inactive during membrane passage.

## Discussion

The experiments described in this paper show that *B. amyloliquefaciens* AprE is responsible for activating mersacidin in a second proteolytic step, which occurs after transport and partial leader processing by MrsT. While this kind of leader processing has been described previously, for example, subtilin (Corvey et al., 2003), this result supports the notion that RiPP leader removal by proteases outside the biosynthetic gene cluster may be more common than previously thought (Völler et al., 2013; Chen et al., 2019).

The identification of AprE as the responsible protease for mersacidin leader removal, and its production in *E. coli*, greatly enhances the applicability of the previously described heterologous expression system for the mersacidin biosynthetic gene cluster (Viel et al., 2021). Like the *B. subtilis* ATCC 6633 AprE (van Tilburg et al., 2020), *B. amyloliquefaciens* AprE can be tagged with a C-terminal His-tag, allowing for convenient purification. While heterologous expression of AprE from another *B. amyloliquefaciens* strain has been shown previously (Wells et al., 1983; Bjerga et al., 2016), the results shown in this study go beyond a proof of principle and prove its usefulness in the biosynthesis of RiPPs.

The observed ability of BH072 AprE to activate mersacidin compared to ATCC 6633 AprE is notable, and the sequences of both proteases were compared to identify a possible cause. Both homologs are expressed as a longer protein, which are autoprocessed into a shorter active enzyme (Figure 3; Ikemura et al., 1987; Ohta and Inouye, 1990). The sequence similarity between the homologs is lowest (75%) in the N-terminal export signal pre-sequence, which could explain the lower yield of BH072 AprE in the PG10 supernatant. The C-terminal sequences that make up the active enzyme have 86% similarity. However, no notable differences between the amino acid sequences were found regarding conserved catalytic triad residues (Carter, 1988; Supplementary Material S2), residues forming the S_1_ and S_4_ hydrophobic pocket (Czapinska and Otlewski, 1999), or residues known to be involved in substrate specificity, e.g., Gly127 Gly166 (Takagi et al., 1996; Matsumoto et al., 2001; Supplementary Material S2). The difference in mersacidin leader removal efficiency between these AprE homologs does not seem to have straight forward explanation and requires more in dept. research to elucidate.

In a synthetic biology setting, removing the mersacidin leader from modified precursor peptides using His-AprE has many advantages over digestion with BH072 supernatant. Purification and analysis of digested products are more straightforward and require less additional workup. Furthermore, the absence of other supernatant proteases allows for an analysis of the proteolytic steps performed by AprE and to make design improvements where necessary. As is well established and confirmed here, the properties of AprE itself are also very convenient. It is well produced in both *E. coli* and *Bacillus* strains and is quite resistant to degradation. However, since AprE has a broad substrate specificity (Carter et al., 1989), one should be careful with the application of AprE to release linear, or partially modified, peptides.

The MALDI-TOF analysis of fully modified and partially modified His6-MrsA digests by the different ATCC 6633 proteases gives an interesting insight into the specificity of the tested proteases. AprE is the only protease produced by *B. subtilis* 168 able to fit amino acids, that are part of an intramolecular ring, into its active site, and efficiently perform a proteolytic step. Bpr, Epr, Vpr, and WprA from ATCC 6633 were only able to cleave the mersacidin leader when the core peptide was not fully modified. Interestingly, this experiment concomitantly gave insight into the maturation process of mersacidin. MALDI-TOF analysis of MrsMD modified His6-MrsA produced in *E. coli* shows that most product is fully dehydrated (Viel et al., 2021). However, the closing of ring A seems to be a limiting factor when producing active mersacidin, which partially explains the shift that is observed in HPLC spectra of modified His6-MrsA.

Taking the previous into account, *B. subtilis* 168 *ΔaprE* could prove to be an interesting industrial expression host for mersacidin. Using only the biosynthetic genes *mrsAMDT*, fully modified GDMEAA-mersacidin should be transported out of the cell, while remaining inactive until a final digestion step with AprE. Under the right conditions, higher yields might be achievable using this system than is reported from the natural producer (Bierbaum et al., 1995). Furthermore, preventing immediate activity upon production might prove useful in certain other applications, like inhibition or detection of *Bacillus* strains, using *B. subtilis* 168 *ΔaprE* as in indicator strain in combination with fully modified mersacidin precursor peptide to detect AprE producing *Bacillus* strains. Finally, it is possible that an AprE knockout strain of *B. amyloliquefaciens* gives better mersacidin yields than *B. subtilis* 168 *ΔaprE*, but at this moment, it cannot be excluded other proteases produced by *B. amyloliquefaciens* can activate mersacidin as well.

The results from this paper indicate that the last six amino acids of the leader probably play no beneficial role after the core peptide has been fully modified, as no effect on diffusion or antimicrobial activity can be observed between presence and absence of leader peptide fragments. One could argue that keeping mersacidin inactive upon transport prevents the auto-induction mechanism from activating. However, due to the nature of this mechanism, this does not seem beneficial. Mersacidin expression is regulated by the growth phase of the producer, and the production can be non-dose-dependently advanced to an earlier growth stage by the addition of a relatively high concentration of mersacidin (Schmitz et al., 2006). Thus, the production does not rely on accumulation of low-level constitutive expression as is the case in lanthipeptides like nisin (Mierau and Kleerebezem, 2005). Additionally, since overnight *B. amyloliquefaciens* supernatant can activate mersacidin (Viel et al., 2021), the production of AprE starts earlier than that of mersacidin (Bierbaum et al., 1995), meaning mersacidin will most likely be readily activated upon its transport out of the cell. Another possibility is that keeping mersacidin inactive during transport may facilitate membrane passage and diminishes any possible toxic effects immediately after transport. A shorter leader during transport might also facilitate the secretion of the molecule over the membrane.

Since all class II lanthipeptide transporters have a transporter and proteolytic domain (Altena et al., 2000; Arnison et al., 2013; Bobeica et al., 2019; Kieuvongngam et al., 2020), cleaving of the leader peptide probably plays an important role in the complete mode of action behind mersacidin export. If ring A of mersacidin indeed inhibits leader processing by most tested proteases, the six amino acids downstream of the conserved class II lanT cleavage site could play a buffering role in the efficient cleavage and transport of the mersacidin precursor peptide. However, in lacticin 3,147 the leader is cleaved completely by LtnT even though there a similar ring structure, albeit CS instead of CT, is in place (Altena et al., 2000; Suda et al., 2012). The first ring in mersacidin is, however, directly followed by a large seven amino acid ring. It is possible that the more rigid structure formed by ring A and B together is difficult for proteases to cleave.

## Conclusion

The identification and production of the mersacidin leader protease AprE greatly improve the heterologous expression system for mersacidin previously reported on (Viel et al., 2021), while revealing the full two-step proteolytic activation steps of mersacidin when produced naturally. Additionally, the functional necessity of the two-step leader processing is found to be most likely related to the correct modification and secretion efficiency of mersacidin.

## Data Availability Statement

The original contributions presented in the study are included in the article/**Supplementary Material**, further inquiries can be directed to the corresponding author.

## Author Contributions

Experiments were conceived and designed by JV and OK and then performed by JV. *Bacillus subtilis* proteases and knockout strain were provided by AT. Results were analyzed by JV and OK. The paper was written by JV. All authors contributed to reading and correcting the paper.

## Funding

JV was funded by the Netherlands Organization for Scientific Research (NWO, ALWOP. 214). AT was supported by a grant from European Union’s Horizon 2020 Research and Innovation Program (Grant Agreement No. 720776).

## Conflict of Interest

The authors declare that the research was conducted in the absence of any commercial or financial relationships that could be construed as a potential conflict of interest.

## Publisher’s Note

All claims expressed in this article are solely those of the authors and do not necessarily represent those of their affiliated organizations, or those of the publisher, the editors and the reviewers. Any product that may be evaluated in this article, or claim that may be made by its manufacturer, is not guaranteed or endorsed by the publisher.
