# Supplementary material for: Characterization of Leader Processing Shows That Partially Processed Mersacidin Is Activated by AprE After Export
Source: Front Microbiol. 2021 Oct 28;12:765659. doi: 10.3389/fmicb.2021.765659 (PMC8581636; doi:10.3389/fmicb.2021.765659)
Supplement: Supplementary file 1 [file Data_Sheet_1.docx]

**Supplementary material**

[S1. Digestion of (pre-HPLC) MrsMD modified His6-MrsA by ATCC 6633 proteases MALDI-TOF spectra 2](#_Toc83728854)

[S2. A comparison of ATCC 6633 and BH072 AprE 3](#_Toc83728855)

[S3. Tricine gel and western blot of AprE-His production 8](#_Toc83728856)

[S4. Purification of His-mersacidin leader peptide 9](#_Toc83728857)

[S5. List of primers used in this study 10](#_Toc83728858)

[S6. Verification of digestion for diffusion tests by MALDI-TOF analysis 11](#_Toc83728859)

# S1. Digestion of (pre-HPLC) MrsMD modified His6-MrsA by ATCC 6633 proteases MALDI-TOF spectra

**
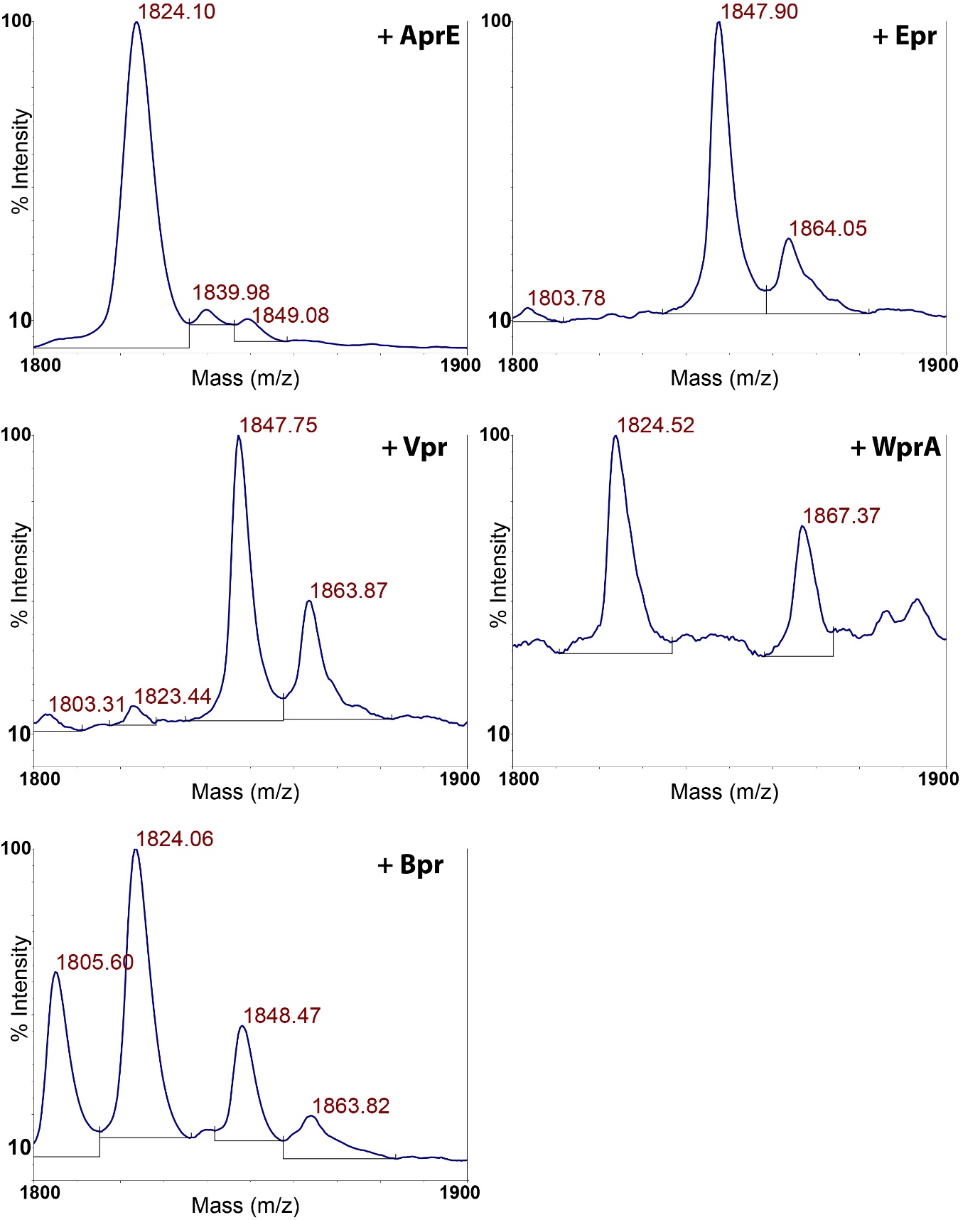
**After digestion of (pre-HPLC) modified His6-MrsA by the respective ATCC 6633 proteases, a mass resembling that of mersacidin (1826 Da) was found in the samples digested by AprE, Bpr, WprA and Vpr. However, only AprE digested modified His6-MrsA had antimicrobial activity.

# S2. A comparison of ATCC 6633 and BH072 AprE

The aprE homolog of *B. amyloliquefaciens* BH072 was found by blasting amino acid sequence resulting from the GenBank AJ539133.1 nucleic acid sequence against the BH072 genome. This led to the identification of the GenBank: CP009938.1 “Peptidase S8” gene.

**Genetic information**

Bacillus subtilis ATCC 6633 *aprE* “*aprE gene* for subtilisin precursor”
GenBank: AJ539133.1

GTGAGAAGCAAAAAATTGTGGATCAGCTTGTTGTTTGCGTTAACGTTAATCTTTACGATGGCGTTCAGCAACATGTCTGCGCAGGCTGCCGGAAAAAGCAGTACAGAAAAGAAGTACATTGTCGGATTTAAACAGACAATGAGTGCGATGAGTTCCGCCAAGAAAAAGGATGTTATTTCTGAGAAAGGCGGAAAAGTCCAAAAGCAGTTTAAGTATGTTAACGCAGCCGCAGCAACATTGGATGCAAAAGCAGTAAAAGAATTGAAACAAGATCCGAGCGTTGCATATGTGGAAGAAGACCATATTGCACATCAATATGCGCAATCCGTTCCTTACGGCATTTCTCAAATTAAAGCGCCGGCTCTTCACTCTCAAGGCTACACAGGTTCTAACGTAAAAGTAGCCGTAATTGACAGCGGAATTGACTCTTCTCATCCTGACTTGAACGTCAGAGGCGGAGCAAGCTTCGTACCTTCTGAAACAAACCCATACCAAGATGGCAGTTCTCACGGCACACATGTAGCCGGTACGGTTGCCGCACTTAATAACTCAATCGGTGTTTTGGGCGTAGCGCCAAACGCATCGTTATATGCAGTAAAAGTTCTTGATTCAACAGGAAACGGCCAATACAGCTGGATTATTAACGGCATTGAGTGGGCCATTTCCAACAAAATGGACGTGATTAACATGAGCCTTGGCGGACCTTCTGGTTCTACAGCTTTGAAATCAGTCGTTGATAGAGCCGTAGCCAGCGGTATCGTCGTTGTTGCTGCAGCCGGAAATGAAGGCACTTCCGGAAGCTCAAGCACAATCGGCTATCCTGCAAAATATCCTTCTACCATTGCGGTAGGTGCGGTAAACAGCAGCAACCAAAGAGGTTCATTCTCAAGCGTAGGTCCTGAGCTTGATGTAATGGCTCCTGGTGTATCCATCCAAAGCACACTACCTGGAGGCACTTACGGCGCTTACAACGGAACGTCAATGGCGACTCCTCACGTTGCCGGAGCAGCAGCGTTAATTCTTTCTAAGCACCCGACTTGGACAAACGCACAAGTCCGTGATCGTTTAGAAAGCACTACAACATACCTTGGAAACTCTTTCTACTATGGAAAAGGGTTAATCAACGTACAAGCAGCTGCACAATAA

Bacillus Amyloliquefaciens BH072 *AprE* “Peptidase S8”
GenBank: CP009938.1

CTGAGCTGCCGCCTGTACGTTGATCAGCCCTTTTCCGTAGTAGAAAGCATCACCAAGTTTTGTAGTGGTGTTTTCTAAACTGCTGCGGACTTGAGTGTTTGTCCAGTTCGGGTGCTTAGAAAGAATCAAAGCAGCCGCTCCGGCAACGTGCGGAGATGCCATTGACGTACCATTGTACGCGCCGTATTTGTTTCCAGGAAGCGTGCTTTGGATAGAGACGCCTGGTGCCATGACATCAAGCTCAGAACCTACGCTTGAGAAAGATGCTCTTTGGTTGCTGCTGTTAACTGCCCCTACCGCAATGACAGAAGGGTATTTACCAGGGTAGCCCACTGTGCTTGAGCTGCCGGAAGTGCCTTCGTTACCGGCTGCCGCTACGACTACGATGCCGGAAGCAACGGCTTTGTCAACTGCCGCTTTTAACGCTGCAGAACCAGAAGGTCCGCCGAGGCTCATGTTAATAACGTCCATATTGTTTGCGATCGCCCACTCAATTCCGTTAATGATCCAGCTGTACTGGCCGGAACCGTCAGCGCCGAGAACTTTTACAGCGTAAAGAGATGCGCTTGGCGCAACGCCTAATACACCGACTGAGTTATTAAGAGCCGCAACTGTACCGGCAACGTGAGTTCCGTGAGAGTTGTTGTCTTGGAAAGGATTTGTTTCAGAAGGAACCATGCTGGCTCCGCCTGCTACCTTTAAATCAGGATGAGAAGAATCGATACCGCTGTCGATAACCGCTACTTTAACATTTGATCCGGTGAAGCCTTGAGAGTGCAGAGCAGGGGCTTTAATCTGTGATACGCCGTAAGGCACGGACTGCGCGTACGCCTGTGCAACGTGATCTTCTTCAACGTAAGCGACGCTAGGGTCTTTTTTCAGCTCTTTTACAGCTTTTTCATTTAATGTAGCTGAAGCTGCGTCTACATATTTGAATTGCTTTTGCACTTTCCCGCCTTTTTCAGAAATGACATCTTTTTTCTTAGCGGCGCTCATCGTGCTCATTGTCTGTTTAAATCCGACAATGTATTTCTTTTCCCCGTTTGATTTCCCTGCCGCCTGGGCAGGAGACGTGCTGCCGAACGCCATCGTAAAGATTAACGCTAAAGCAAACAGCAAACTGATCCATACCTTTTTGCCTCTCAC

**Alignment of translated nucleotide sequences**


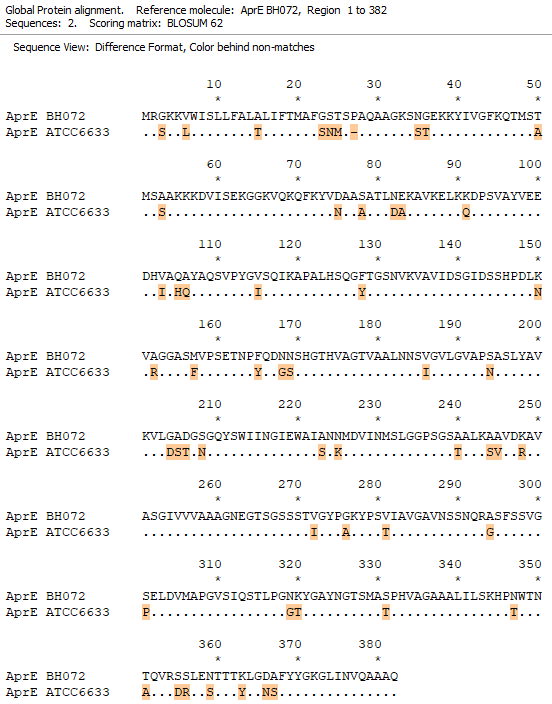


The AprE homolog (peptidase S8) has 85 % sequence similarity (327 Match, 55 NonMatch) to AprE from ATCC 6633

**Alignment of expressed His-tagged proteins**

**
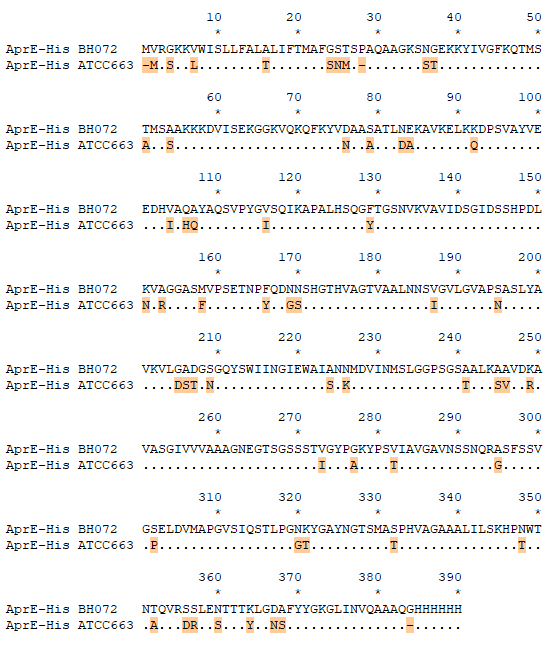
**

The aligned non-auto-processed amino acid sequences of the His-tagged AprE genes from BH072 and ATCC 6633, as they were expressed with a C-terminal His-tag. The calculated average masses are 40117.6 Da (BH072) and 40376.8 Da (ATCC 6633)

**Alignment and analysis of different regions**


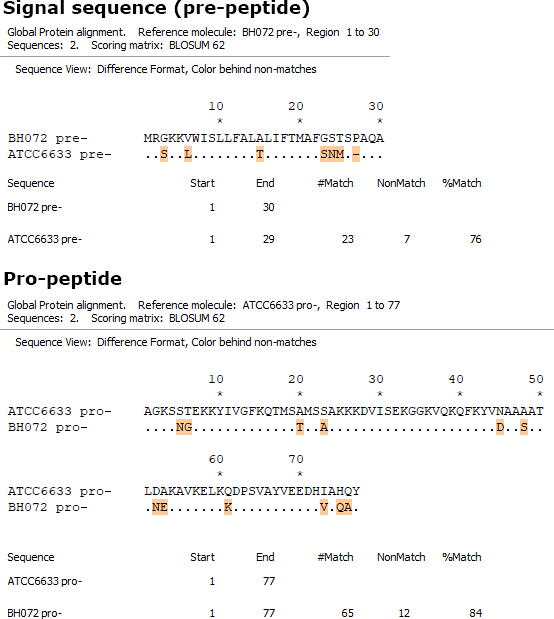
The first ca. 29 residues of ATCC6633 and BH072 AprE, which make up the signal sequence for export(Ikemura et al., 1987), have the lowest sequence similarity (76 %). The pro-peptide sequence is more conserved at 84 % sequence similarity.

**
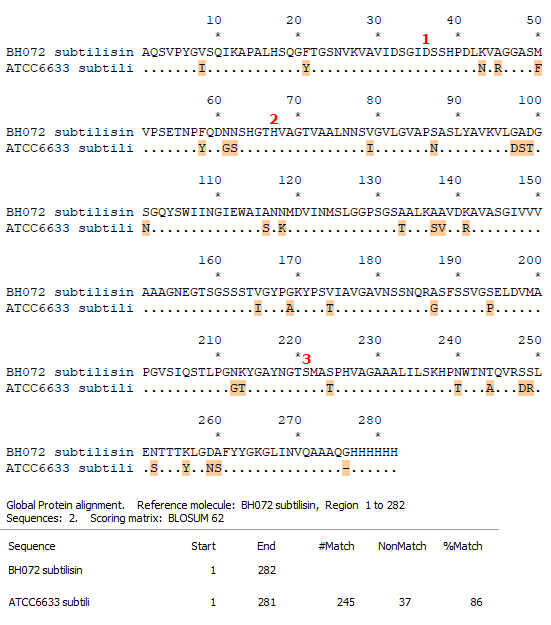
** **Fully autoprocessed C-terminal proteolytic sequence (Subtilisin E)**

The aligned predicted auto-processed amino acid sequences (based on uniport entry P04189 (SUBT_BACSU)) of the His-tagged AprE genes from BH072 and ATCC 6633. Predicted auto-processed masses are 28370.1 Da (BH072) and 28655.3 Da (ATCC 6633). The red numbers are over the well-established conserved residues of the subtilisin catalytic triad (D32, H64, S221)(Carter, 1988; Ekici et al., 2008).

# S3. Tricine gel and western blot of AprE-His production

**
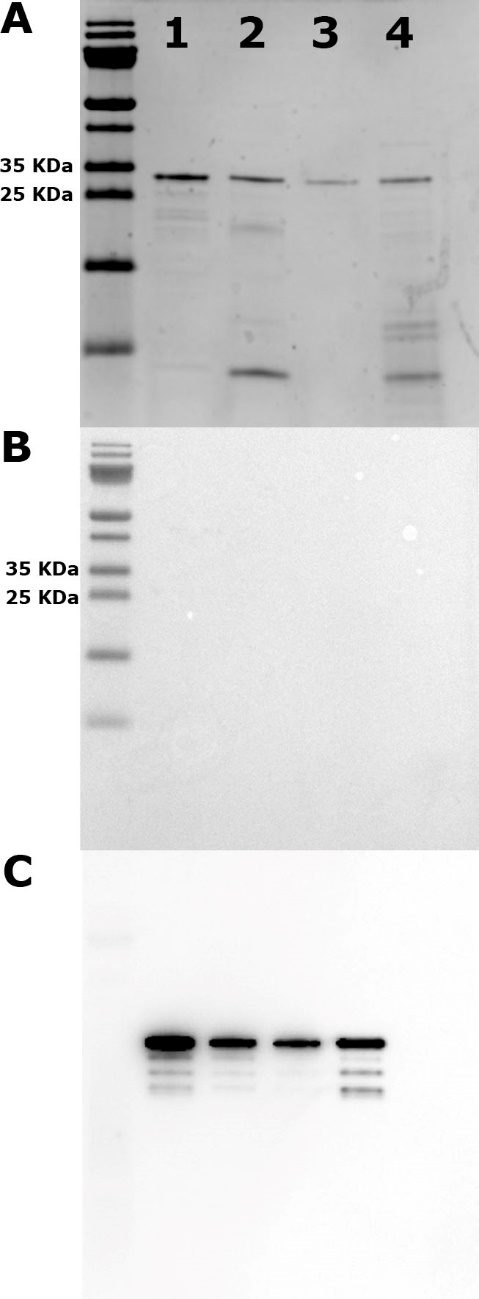
**The tricine gel (A) and western blot (B, C), run as described in the main text. To compile the western blot image, the PageRuler on the epi-white image of the western blot (B) was overlaid on the chemiluminescence image (C). Samples: (1) ATCC 6633 AprE-His (PG10), (2) ATCC 6633 AprE-His (*E. coli*), (3) BH072 AprE-His (PG10), (4) BH072 AprE-His (*E. coli*).

# S4. Purification of His-mersacidin leader peptide

**
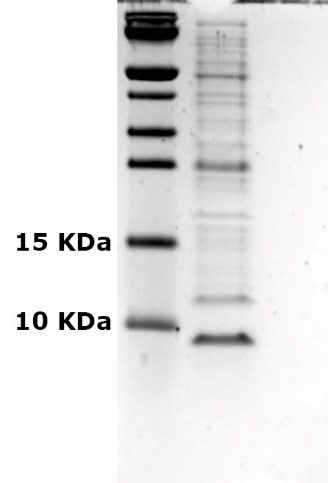
**
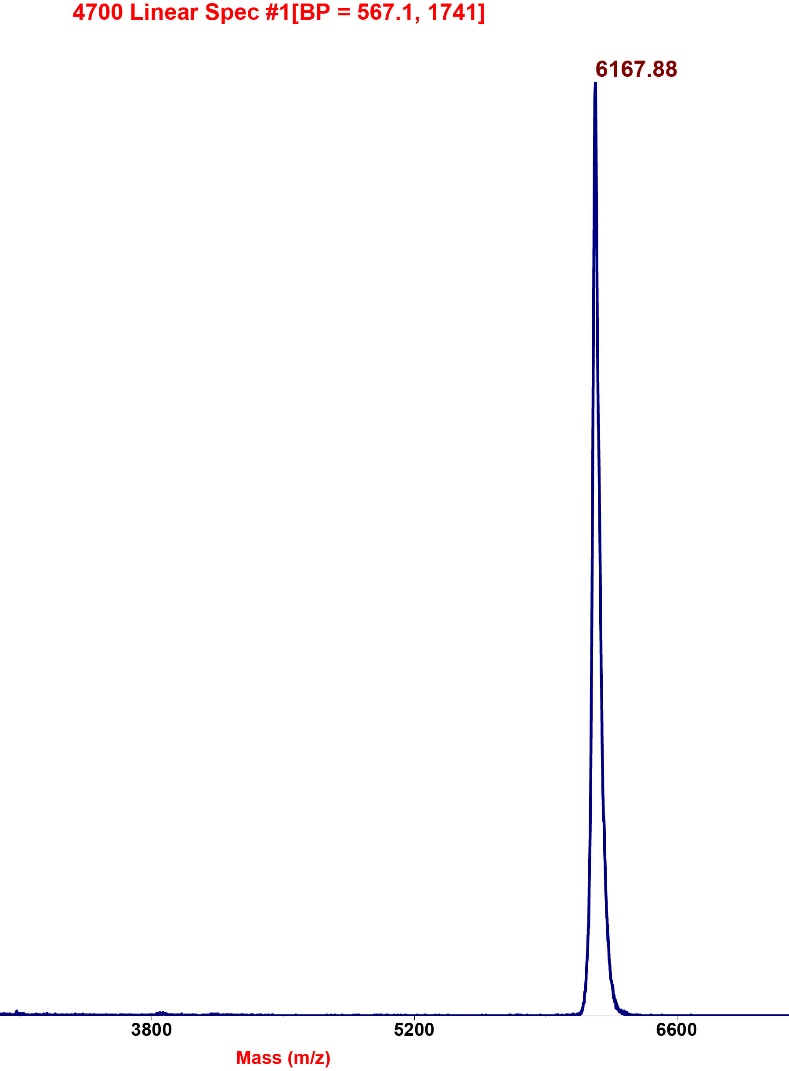
The tricine gel image of His-tag purified His-mersacidin leader peptide and MALDI-TOF analysis of the C18 purified sample. The peptide (MHHHHHHGSQEAIIRSWKDPFSREN
STQNPAGNPFSELKEAQMDKLVGAGDMEAA) has a predicted mass of 6163,7 Da. The amount brought on gel was from ca. 1.5 mL of expression medium. The measured mass by MALDI-TOF analysis approximates the predicted mass, confirming its successful production.

# S5. List of primers used in this study

| Primer | Function | Template | Result |
| --- | --- | --- | --- |
| GACGGGTCTCCAGAGGCAAAAAGGTATGGATCAG | Insert fw | pACYC AprE-His (BH072) | pDR111 AprE-His (BH072) |
| GACGGGTCTCTGGTGATGATGCTGAGCTGCCGCCTGTACGTTG | Insert rv | pACYC AprE-His (BH072) |  |
| GACGGGTCTCGCACCATCATCACTAAGATAAAAAAGAAGCAGGCATG | Backbone fw | pDR111 AprE-His (ATCC 6633) |  |
| GACGGGTCTCGCTCTCATTCTTTACCCTCTCCTTTTAG | Backbone rv | pDR111 AprE-His (ATCC 6633) |  |
| GACGGGTCTCAATGAGAAGCAAAAAATTGTGGATCAGC | Insert fw | pDR111 AprE-His (ATCC 6633) | pACYC AprE-His (ATCC 6633) |
| GACGGGTCTCGGATGTTGTGCAGCTGCTTGTACGTTG | Insert rv | pDR111 AprE-His |  |
| GACGGGTCTCGCATCATCACCATCATCACTAATTTAAGC | Backbone fw | pACYC MrsA-His |  |
| GACGGGTCTCCTCATGGTATATCTCCTTATTAAAGTTAAAC | Backbone rv | pACYC MrsA-His |  |
| CAGCGGTCTCACATGGTGAGAGGCAAAAAGGTATGG | Insert fw | BH072 genomic DNA | pACYC AprE-His (BH072) |
| GAGCGGTCTCGGACCCTGAGCTGCCGCCTGTACGTTG | Insert rv |  |  |
| GAGCGGTCTCCGGTCATCATCACCATCATCAC | Backbone fw | pACYC MrsA-His |  |
| CATGGGTCTCCCATGGTATATCTCCTTATTAAAGTTAAAC | Backbone rv | pACYC MrsA-His |  |
| GACGGGTCTCCTAATTTAAGCTTTCTTTGAACCCTGCAGC | Round fw | pACYC His-MrsA | pACYC His-mersacidin leader |
| GACGGGTCTCCATTATGCTGCTTCCATGTCTCCCG | Round rv | pACYC His-MrsA |  |
| CGTAGGCCAACGCGGCCGTTTTGCCCTGCATAATCCTTTG | *aprE* up fw | *B. subtilis* 168 genomic DNA | pJOE_ ΔaprE vector |
| CGTAGGCCGAACTGGCCTCTCATTCTTTACCCTCTCC | *aprE* up rv |  |  |
| GCTAGGCCAGTTCGGCCAAAGAAGCAGGTTCCTCCATAC | *aprE* down fw |  |  |
| CGTAGGCCGTATTGGCCATTCAGCCGGTCATTATCGATCC | *aprE* down rv |  |  |

# S6. Verification of digestion for diffusion tests by MALDI-TOF analysis

**
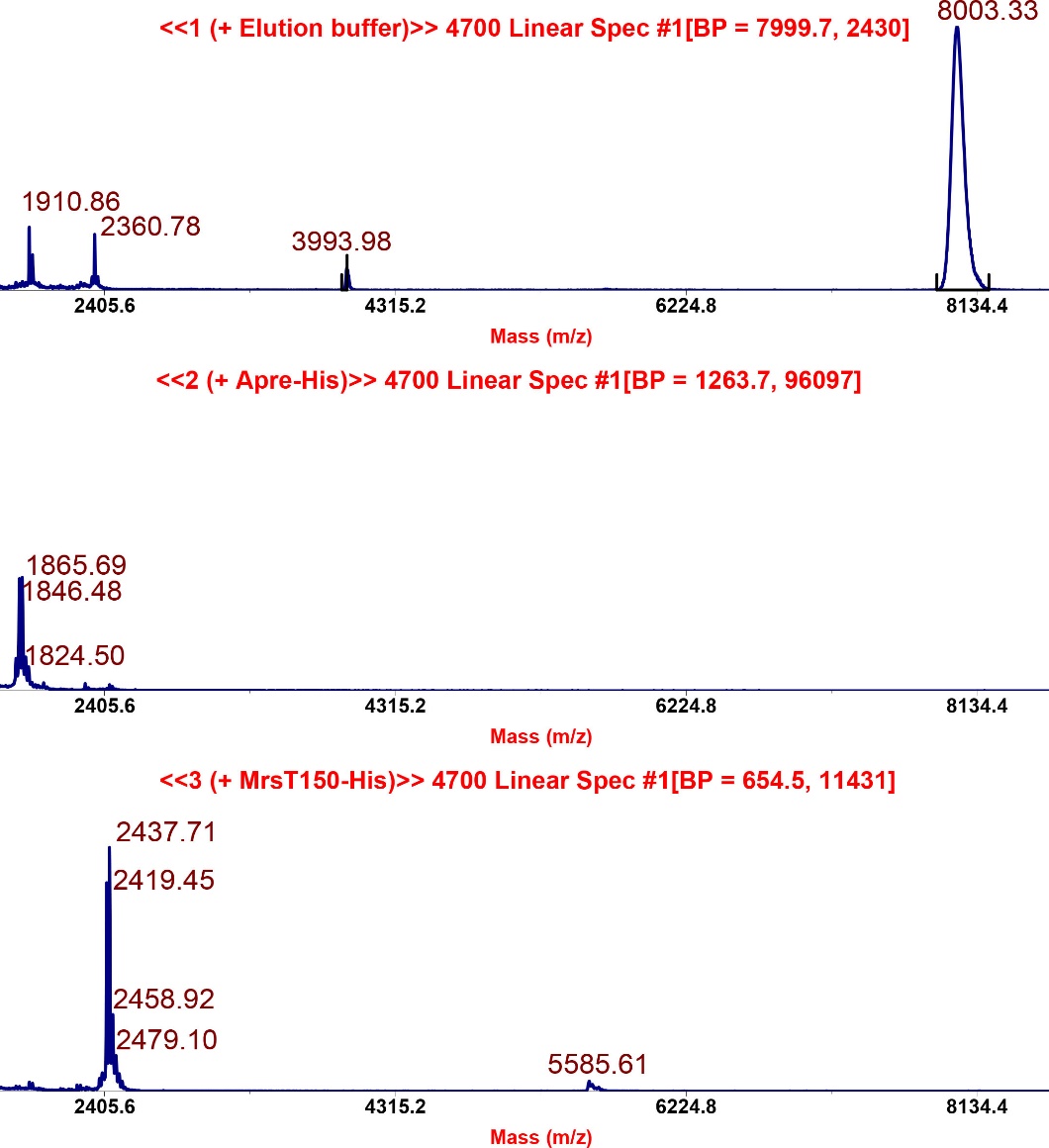
**
MALDI-TOF analysis of the HPLC purified fully modified His6-MrsA digestion for the diffusion tests confirms the prepeptide incubated with elution buffer remains largely intact, the AprE-His digested fully modified His6-MrsA is fully digested, and contained peaks resembling the mass of fully modified mersacidin (1824.50 Da, 1826 Da theoretical) and those of mersacidin + Na (1846.48 Da, 1848 Da theoretical) and + K (1865.69 Da, 1864 Da theoretical)(Herzner et al., 2011). The MrsT150-His digested fully modified His6-MrsA is fully digested, and contains the expected masses in regard to the MrsT cleavage site(Viel et al., 2021), likely +Na and +K GDMEAA-mersacidin (2401.84 Da average theoretical mass), and the remaining leader peptide at 5585.61 Da (5590.10 Da average theoretical mass) . These measurements function as a control for a representative diffusion-assay of partially cleaved pre-mersacidin.

**References**

Carter, P. A. J. A. W. (1988). Dissection of Catalytic Triad of Serine Proteases. *Nature* 332, 564–568.

Ekici, Ö. D., Paetzel, M., and Dalbey, R. E. (2008). Unconventional serine proteases: Variations on the catalytic Ser/His/Asp triad configuration. *Protein Sci.* 17, 2023–2037. doi:10.1110/ps.035436.108.

Herzner, A. M., Dischinger, J., Szekat, C., Josten, M., Schmitz, S., Yakéléba, A., et al. (2011). Expression of the Lantibiotic Mersacidin in *Bacillus amyloliquefaciens* FZB42. *PLoS One* 6, e22389. doi:10.1371/journal.pone.0022389.

Ikemura, H., Takagi, H., and Inouye, M. (1987). Requirement of pro-sequence for the production of active subtilisin E in *Escherichia coli*. *J. Biol. Chem.* 262, 7859–7864.

Viel, J. H., Jaarsma, A. H., and Kuipers, O. P. (2021). Heterologous Expression of Mersacidin in *Escherichia coli* Elucidates the Mode of Leader Processing. *ACS Synth. Biol.* 10, 600–608. doi:10.1021/acssynbio.0c00601.
